# Calcium Oxalate Crystals in Eucalypt Ectomycorrhizae: Morphochemical Characterization

**DOI:** 10.1371/journal.pone.0067685

**Published:** 2013-07-02

**Authors:** Victor Satler Pylro, André Luiz Moreira de Freitas, Wagner Campos Otoni, Ivo Ribeiro da Silva, Arnaldo Chaer Borges, Maurício Dutra Costa

**Affiliations:** 1 Departamento de Microbiologia, Universidade Federal de Viçosa, Viçosa, Minas Gerais, Brazil; 2 Departamento de Biologia Vegetal, Universidade Federal de Viçosa, Viçosa, Minas Gerais, Brazil; 3 Departamento de Solos, Universidade Federal de Viçosa, Viçosa, Minas Gerais, Brazil; Cinvestav-IPN, Mexico

## Abstract

Ectomycorrhizal fungi are ubiquitous in forest ecosystems, benefitting plants principally by increasing the uptake of water and nutrients such as calcium from the soil. Previous work has demonstrated accumulation of crystallites in eucalypt ectomycorrhizas, but detailed morphological and chemical characterization of these crystals has not been performed. In this work, cross sections of acetic acid-treated and cleared ectomycorrhizal fragments were visualized by polarized light microscopy to evaluate the location of crystals within cortical root cells. Ectomycorrhizal sections were also observed by scanning electron microscopy (SEM) coupled with energy dispersive x-ray (EDS) microprobe analysis. The predominant forms of crystals were crystal sand (granules) and concretions. Calcium, carbon and oxygen were detected by EDS as constituent elements and similar elemental profiles were observed between both crystal morphologies. All analyzed crystalline structures were characterized as calcium oxalate crystals. This is the first report of the stoichiometry and morphology of crystals occurring in eucalypt ectomycorrhizas in tropical soils. The data corroborates the role of ectomycorrhizae in the uptake and accumulation of calcium in the form of calcium oxalate crystals in hybrid eucalypt plants.

## Introduction

The occurrence of calcium oxalate crystals (CaC_2_O_4_ or CaOx) has been observed in plants of several botanical families [1], and they contribute a large portion of the total calcium of these plants. CaOx deposits have been described in most tissues and organs as either intracellular (usually associated with vacuoles of specialized cells; idioblasts) or extracellular [1]–[4] deposits. The biological function of CaOx crystals in plants is neither completely understood nor characterized. Several functions have been attributed to them, largely based upon the amount, distribution and morphology of the crystals as well as the inherent characteristics of the cells where they are produced [1]. Some studies imply that CaOx may serve different biological functions such as a calcium reservoir, deposit of secondary metabolites and sequestration of potentially toxic metal ions [5], [6], formation of aerenchyma in aquatic plants [7], providing structural support [8], or protection against herbivory by association with stinging substances or proteolytic toxins [9]–[11].

*Eucalyptus* is the most important genus of exotic plants in Brazilian planted forests, with great economic and environmental significance [12]. Generally, the soils under eucalypt cultivation in Brazil are highly weathered, with pH values below 5.5, an Aluminum saturation of 90%, and a low content of organic matter and limiting concentrations of Phosphorus, Nitrogen and Calcium (Ca^2+^) [13]–[16]. Soil exchangeable calcium is often at or below 8 kg ha^−1^ and insufficient to fulfil the plant demand [17]. Soil microorganisms play an essential role in biochemical cycles and contribute to edaphic homeostasis. In the current understanding that biological and functional diversity is a crucial factor in maintaining ecosystems [18] are included ectomycorrhizal fungi associated with the roots, that benefit plants by increasing the volume of the soil explored by roots, and thus the amount of absorbed nutrients and water [19].

The presence of CaOx crystals in ectomycorrhizal hyphae is limited to temperate soils containing high concentrations of calcium [20]–[22]. In Brazil, the number of studies aiming at evaluating the accumulation of calcium crystals in eucalypt ectomycorrhizae is scarce [23]. However, the existing report has suggested a paramount role of ectomycorrhizal fungi in supplying Ca to eucalyptus in Brazilian soils poor in Ca, since putative CaOx crystals have been shown to be predominantly present in ectomycorrhizae rather than in non-mycorrhizal fine roots. Understanding the morphochemical patterns of crystalline structures in *Eucalyptus* ectomycorrhizae can help generate new information regarding the role of ectomycorrhizal fungi in Ca^2+^ uptake by plants. Therefore, the aim of this study was to evaluate the location, morphology and chemical composition of crystals present in eucalypt ectomycorrhizae.

## Materials and Methods

The experiments were performed at Mycorrhizal Associations Laboratory/BIOAGRO, Microbiology Department of Federal University of Viçosa (UFV). Analyses of Scanning Electron Microscopy (SEM) and Energy Dispersive X-ray (EDS) were performed at the Microscopy Center of the Federal University of Minas Gerais. All samples were processed at the Center of Microscopy and Microanalysis at UFV.

### Characterization of Sampling Site

Samples were taken from within a 2.5 year old planted stand of clonal *E. grandis* X *E. urophylla* hybrids located in the experimental field of UFV (20° 46′ 27.3′′ S and 42° 51′ 36.2′′ W; elevation: 697 m). Ten soil cores (2.0 cm diameter, 20 cm depth) were collected at random points within the study area, and the cores were combined to form a composite sample. Physicochemical analyses (Table 1) were performed according to routine methods.

**Table 1:** Physicochemical properties of the soil sampled in planting area of *Eucalyptus grandis* X *E. urophylla* hybrid.

Property	Unit	Value
pH– H_2_O (1∶2,5)		5.30
P	mg kg^−1^ (1)	41.70
K	mg kg^−1^ (1)	97.00
Ca^2+^	cmol_c_ dm^−3^ (1)	1.20
Mg^2+^	cmol_c_ dm^−3^ (2)	0.50
Al^3+^	cmol_c_ dm^−3^ (2)	0.20
H+Al	cmol_c_ dm^−3^ (3)	4.95
SEB	cmol_c_ dm^−3^	1.95
ECEC	cmol_c_ dm^−3^	2.15
CEC(T)	cmol_c_ dm^−3^	6.90
BS	%	28.00
ASI	%	9.00
MO	% (4)	3.30
P - remaining	mg L^−1^ (5)	30.60
Zn	mg kg^−1^ (1)	11.10
Fe	mg kg^−1^ (1)	54.20
Mn	mg kg^−1^ (1)	62.90
Cu	mg kg^−1^ (1)	1.20
B	mg kg^−1^ (6)	0.90
Sand	%(7)	59.00
Silt	%(7)	18.00
Clay	%(7)	23.00
Textural class		Sand-clay loam

(1) Extracted with Mehlich-1.

(2) Extracted with KCl 1 mol L^−1^.

(3) Extracted with calcium acetate 0.5 mol L^−1^, pH 7.0.

(4) Walkey & Black method.

(5) P concentration in solution after 1 h shaking with a 60 mg L^−1^ P (1∶10 soil:solution ratio) [51].

(6) Extracted with hot water.

(7) Pipet method. SEB = Sum of Exchangeable Bases. ECEC - Effective Cation-Exchange Capacity. CEC (T) - Cation-Exchange Capacity in pH 7.,0. BS = Base Saturation. ASI = Aluminum Saturation Index.

Fine lateral roots colonized by ectomycorrhizal fungi were collected from the 0–10 cm layer of soil around randomly selected trees within the plantation. The soil was excavated and the roots were collected with fine-tipped forceps and razor blades. Samples were placed in a moist chamber and transported to the laboratory, for analysis.

### Ectomycorrhizal Eucalypt Preparation, Acid Digestion and Image Processing

Ectomycorrhizal samples were washed in phosphate buffer, pH 7.0, to remove soil particles adhered to the surface, and then dehydrated in an ethanol series [10, 30, 50, 70 and 90% ethanol (vol/vol)] for 10 min in each solution and finally kept in 95% ethanol (vol/vol) for 12 h. Subsequently, part of the samples was cleared in a 1.8% (vol/vol) sodium hypochlorite solution for 1 h. The cleared samples containing crystals were treated with 5% acetic acid, followed by three successive rinses in deionized water, to remove calcium carbonates and phosphates before further analyses [24]. The fragments were mounted on semi-permanent slides with a glycerine:gelatin (1∶1) fixative. Slides were viewed and analyzed on an Olympus BX-50® microscope provided with crossed polarizers and a digital image capture system (QColor3® Olympus®). The obtained images were processed with QCapture® Suite Pro 6.0 software (Quantitative Imaging Corp., British Columbia, Canada) and organized with Adobe Illustrator CS5 software (Adobe Systems Incorp., USA).

Ectomycorrhizae samples were mounted in Jung Tissue Freezing Medium® solution and transversely sectioned at -25°C (25–40 µm) using a Leica CM 1850® cryostat. The sections were mounted on semi-permanent slides for microscopic observation.

### Scanning Electron Microscopy and X-ray Microanalysis

Selected ectomycorrhizal fragments were fixed in 2.5% glutaraldehyde in phosphate buffer 1 M, pH 6.8–7.0 (1∶1) for 1 h, and then washed in 1 M phosphate buffer, pH 6.8–7.0 for 10 min., followed by dehydration through an ethanol series [10, 30, 50, 70, 90, 100, and 100% (vol/vol)] at 1 h per step. Samples were CO_2_ critical-point dried (Balzers CPD 030), mounted on Aluminum SEM stubs and sputter coated with ca. 15 nm Gold (Balzers SCA 010). Samples were observed by scanning electron microscope (Quanta 200 series FEG, FEI™), equipped with Energy Dispersive X-Ray Analysis Pegasus integrated EDS, for chemical analysis. The operating conditions were 10, 15 or 30 kV, 750 mA, and scanning time of 30 s.

## Results

### Distribution and Morphology of Calcium Crystals in Ectomycorrhizal Eucalypts

**Figure 1:**
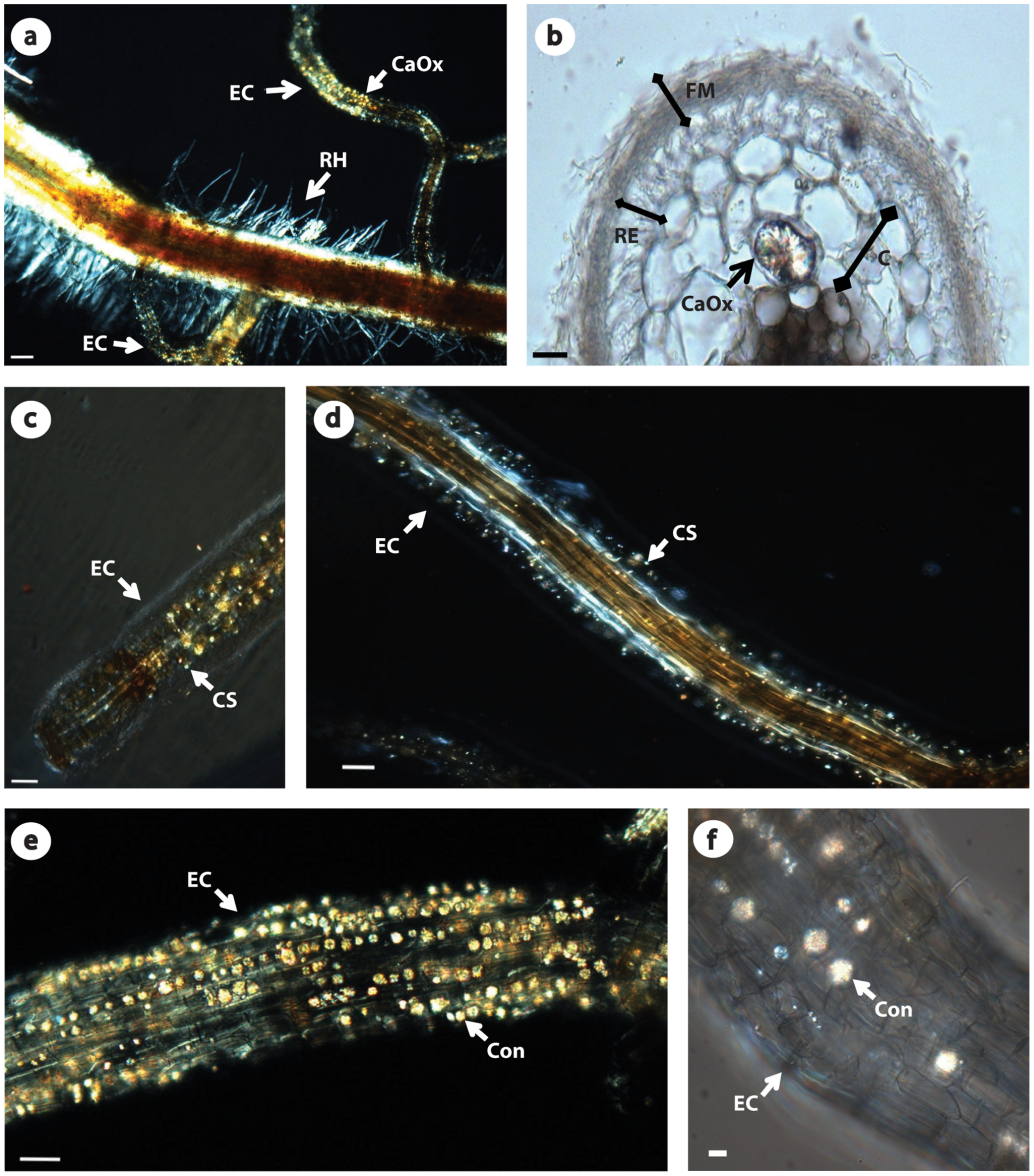
Polarized light micrographs of eucalypt ectomycorrhizae after clarification. (a) Ectomycorrhizal and non-colonized fine lateral roots of *E. grandis* X *E. urophilla* hybrids. Calcium oxalate crystals (CaOx) in ectomycorrhizae (EC), and root hairs (RH) in non-colonized fine lateral roots. Scale bar = 100 µm. (b) Transverse section of ectomycorrhizae. Root cortex (C), root epidermis (RE), mycorrhizal mantle (M) and calcium oxalate crystal (CaOx) within a root cortex cell. Scale bar = 20 µm. (c–d) Crystal sand (CS) calcium oxalate in ectomycorrhizal fragments (EC). Scale bar = 50 µm. (e–f) Concretion (Con) calcium oxalate crystal in ectomycorrhizal fragments (EC). Scale bar = 50 and 10 µm, respectively.

Polarized light microscopy reveals more dense accumulations of crystals associated with ectomycorrhizae compared to non-colonized fine lateral roots of *E. grandis* X *E. urophylla* hybrids (Figure 1a). Transverse sections of ectomycorrhizae revealed that the crystals are formed within root cortical parenchyma cells, and may occupy considerable intracellular volume (Figure 1b).

The acetic acid treatment retained all crystal types observed, indicating calcium oxalate. Two different morphologies were found to be dominant: granules, also known as crystal sand (CS) and spherical crystals, recently named as concretions (Figures. 1c, 1d, 1e and 1f). CS accumulations are easily visualized by polarized light microscopy as bright dots associated with the roots (Figures 1c and 1d). These crystals had sizes ranging from 3 to 8 µm. In contrast, concretions were characterized as large spherical crystals (average size 20 µm) and it could be erroneously classified as druses when observed using crossed polarizers (Figures 1e and 1f). However, druses usually assumes a globular cluster composed of needle shaped crystals, and concretions, despite showing an irregular surface, seem to be comprised of many compacted mini-crystals or CS’s when visualized by scanning electron microscopy (SEM).

### SEM-EDS: Details and Composition of Concretions and Crystal Sand

**Figure 2:**
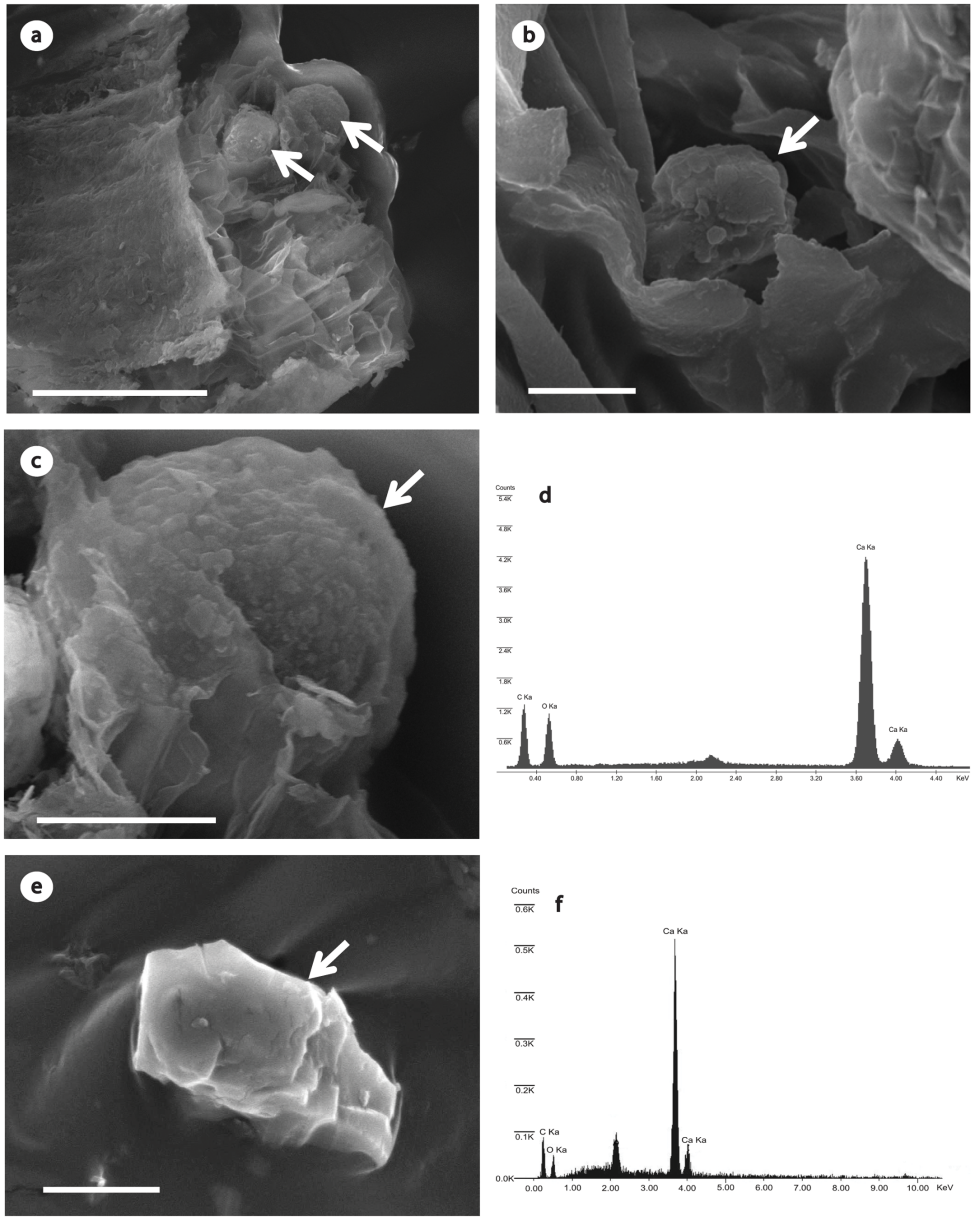
Transverse sections of ectomycorrhizae associated with *E. grandis* X *E. urophilla* hybrids analyzed by SEM. (a) Two calcium oxalate concretions (arrows) located within cortical root cells. Scale bar = 50 µm. (b) Single crystal sand calcium oxalate (arrow) located within cortical root cell. Scale bar = 2 µm. (c) Single concretion analysed by SEM (arrow) showing its irregular surface. Scale bar = 10 µm. (d) Elemental spectrum of Figure 2c (concretion) showing carbon, oxygen and calcium peaks. (e) Single crystal sand analysed by SEM (arrow). Scale bar = 5 µm. (f) Elemental spectrum of Figure 2e (crystal sand) showing carbon, oxygen and calcium peaks.

SEM analyses allowed visualization of the detailed crystal morphology and confirmed the classification as CS and concretions by light microscopy (Fig. 2a and 2b). The x-ray elemental analysis of these crystals showed similar profile for both crystalline types, identifying Calcium, Oxygen and Carbon, confirming a likely calcium oxalate stoichiometry (Figures 2c, 2d, 2e and 2f).

## Discussion

Our results corroborate previous observations that ectomycorrhizal fungi associated with eucalypt fine lateral roots induce accumulation of CaOx crystals in the host plant root cortex [23]. High densities of root hairs were observed in non-colonized fine lateral roots in contrast to ectomycorrhizae-associated roots. The ectomycorrhizal association inhibits the formation of root hairs, which are functionally replaced by fungal hyphae [25]. The inhibitory process involves fungal secretion of indolic compounds such as indole-3-acetic acid and hypaphorine, responsible for root morphogenesis regulation [25]. Generally, hypaphorine leads to root plasma membrane depolarization [26], which should block the elongation of root hairs by decreasing the calcium influx into the tip. However, there are reports of calcium gradient attenuation in root hair cells of *E. globulus,* after treatment with hypaphorine [27]. Therefore, the mechanism of root hair growth inhibition, via mycobiont secreted hypaphorine action, directly influence the flow of Ca^2+^ into root cells.

Previous research regarding eucalypt ectomycorrhizae in calcium-rich soils has identified calcium oxalate crystal crusts on the mantle surface, resultant from Ca^2+^ complexion with oxalic acid produced by the fungi [21], [28]. However, most Brazilian soils used for eucalyptus cultivation are low in calcium content, and perhaps for this reason CaOx crusts were not observed in the mantle, and were only observed within cortical cells in this study. CaOx crystals may show widespread occurrence in various plant organs including roots, stems, leaves, fruits and seeds [29], [30]. Plants exhibit a precise pattern of accumulation of crystals reflecting the multiple levels of control of the crystallization process [31], and its particular distribution and morphology is often used in phylogenetic systematics [32]. It has been suggested that the crystals do not result from random precipitation, where levels of calcium and oxalate are appropriate, but precipitate in certain cells that becomes specialized to accumulate these compounds, called idioblasts [5], [33]. However, crystals may also form less frequently within parenchyma cells or other non-specialized cells [34], as we identified in this study. Crystal accumulation within endosperm of Umbelliferae plants [35] and leaf epidermis of Fabaceae [36] has also been observed. CaOx crystals in the xylem and associated parenchyma cells of *Chamelaucium uncinatum* flowers, a species belonging to the same family as the genus *Eucalyptus* (Myrtaceae) have also been reported [34].

Calcium crystals adopt in a wide range of morphologies [29], [33]. In plants, the most commonly found are prismatic, acicular, crystal sand (granule) or druse crystals [1]. In eucalypt ectomycorrhizae, two crystal forms have been identified by polarized light microscopy: crystal sand, which is characterized as amorphous crystals of uniform size; and druse, defined as clusters of needle-shape crystals forming globular structures [23]. This study, however, shows that this crystalline structure was misclassified as druse. Using SEM we were able to obtain more detailed information regarding the crystal morphology, identifying concretions. This crystal form was recently described in some *Mitragyna* species [24] with probable taxonomic significance in this genus, and previous misidentification of the class of cluster crystals (i.e. druses) was also reported. Similar crystals were observed in Cactaceae and classified as druses in the dihydrate state (Weddellite, CaC_2_O_4_·2H_2_O), while druses formed by needle-shaped crystals as monohydrate (Whewellite, CaC_2_O_4_·H_2_O) [37]. Different plant species typically have varying patterns of crystal morphogenesis that may reflect gene regulation [33]. Franceschi and Nakata [1] reported the combination of genetic and environmental factors was responsible for defining the quantity, shape, size and function of the crystals inside the plants. Differences in nucleation factors, crystal state of hydration, calcium/oxalate ratio, and contaminants can also influence crystal morphology [38], [39]. The biological function of CaOx crystals in plants is poorly understood and the observation of concretions presents a new problem for the understanding of crystal development [24].

The shared stoichiometry of the crystal sands and concretions identified in the eucalypts studied here suggests a common development process. Individual plant species often form only one type of CaOX crystal. This supports the genetic control hypothesis of the crystallization process [1]. Some studies have shown that different crystals can form within the same plant, but this usually occurs in different organs or tissues, or when the plant is subjected to different conditions [40], [41]. High concentrations of calcium induce an increase in the number and size of the crystals, while Ca-deficiency promotes crystal dissolution [6], [42]. Early reports describe the disappearance of CaOx crystals from plants experiencing Ca-deficiency, suggesting remobilization to support growth [43], [44]. However, this has not been verified for ectomycorrhizal CaOx.

Brazilian soils under eucalypt plantations are characteristically low in calcium [17]. Ectomycorrhizal fungi can increase the uptake and translocation of nutrients to the root of the host plant, and this is probably the reason for the elevated accumulation of crystals in roots associated with mycorrhizal fungi. In plants, the Ca^2+^ transport requires strict control, since it plays important roles in cell signaling and metabolism [45], [46]. This control may involve H^+^/Ca^2+^ and Ca-ATPase antiporters, transporting Ca^2+^ to the inner of cytoplasmic vacuoles, endoplasmic reticulum, mitochondria, plastids and cell wall, where calcium can be stored [47]–[49]. However, these homeostatic mechanisms are limited and when overcome can cause damages [42]. Calcium oxalate crystal formation may act as an effective system for sequestering calcium and appears very common in plants [33], [42], [50].

This is the first report concerning the detailed morphology and chemical composition of prevalent crystalline structures in eucalypt ectomycorrhizae in tropical soils. Our results support the proposed role of ectomycorrhizal fungi in the acquisition and translocation of calcium into eucalypt plants, and its strict control being stored as crystals, even in soils with low calcium content conditions. However, the factors and circumstances that modulate the biomineralization process in ectomycorrhizae remain unknown, and further research focused on biochemical and physiological data acquisition is needed.

### Conclusions

Ectomycorrhizae associated with *E. grandis* X *E. urophylla* hybrids accumulate crystals of calcium oxalate within cells of the root cortex. The predominant crystal morphologies are granules and concretions (globular crystals formed by agglomeration of granules). This is the first report of the detailed morphology and chemical composition of crystalline structures in eucalypt ectomycorrhizae.
